# The Influence of Free 3-Nitrotyrosine and Saliva on the Quantitative Analysis of Protein-Bound 3-Nitrotyrosine in Sputum

**Published:** 2007-02-14

**Authors:** Kazuhito Ueshima, Yoshiaki Minakata, Hisatoshi Sugiura, Satoru Yanagisawa, Tomohiro Ichikawa, Keiichirou Akamatsu, Tsunahiko Hirano, Masanori Nakanishi, Kazuto Matsunaga, Toshiyuki Yamagata, Masakazu Ichinose

**Affiliations:** Third Department of Internal Medicine, Wakayama Medical University, Wakayama, Japan.

**Keywords:** 3-nitrotyrosine, high-performance liquid chromatography, free amino acid, saliva, induced sputum

## Abstract

**Background:**

We have recently developed a new technique for quantitatively measuring protein-bound 3-nitrotyrosine (3-NT), a footprint of nitrosative stress, utilizing high-performance liquid chromatography with an electrochemical detection (HPLC-ECD) system. Using this system, we showed that 3-NT formation was upregulated in the sputum of both COPD and asthmatic patients. However, in order to improve the accuracy of the measurement system, We have to resolve some problems which were the influence of free amino acid form of 3-NT and of salivary contamination.

**Objectives:**

We initially investigated the amount of the free amino acid form of 3-NT in induced sputum and compared with that of protein-bound 3-NT. Next, we evaluated the concentration of protein-bound 3-NT in saliva and compared with that in induced sputum by means of HPLC-ECD.

**Methods:**

Five male COPD patients were enrolled. Induced sputum and saliva were obtained from the patients. The free amino acid form of 3-NT in sputum and saliva was measured by HPLC-ECD, and the protein-bound 3-NT and tyrosine in sputum and saliva were enzymatically hydrolyzed by *Streptomyces griseus* Pronase and measured for the protein hydrolysate by HPLC-ECD.

**Results:**

The mean value of the amount of protein-bound 3-NT was 65.0 fmol (31.2 to 106.4 fmol). On the other hand, the amount of the free amino acid form of 3-NT was under the detection limit (<10 fmol). The levels of both 3-NT (sputum: 0.55 ± 0.15 pmol/ml, saliva: 0.02 ± 0.01 pmol/ml, *p* < 0.01) and tyrosine (sputum: 0.81 ± 0.43 μmol/ml, saliva: 0.07 ± 0.04 μmol/ml, *p* < 0.01) in saliva were significantly lower than in sputum. The percentage of 3-NT in saliva to that in sputum was about 3.1%, and that of tyrosine was about 9.0%.

**Conclusion:**

The free amino acid form of 3-NT does not affect the measurement of protein-bound 3-NT. Furthermore, the influence of salivary contamination on the measurement of protein-bound 3-NT in induced sputum by means of HPLC-ECD was very small and could be negligible.

## Introduction

Inflammation of the airways seems to plays an important role in the pathogenesis of chronic obstructive pulmonary disease (COPD) (National Institutes of Health, Updated 2005; Barnes, 2002). However, the pathogenesis has not yet been fully elucidated. Reactive nitrogen species (RNS) may be involved in the pathophysiology of the inflammatory process in COPD (Ichinose et al. 2000; Barnes, 2000). RNS are formed from the reaction of nitric oxide (NO) and superoxide anion (Beckman et al. 1990), or via the H_2_O_2_/peroxidase-dependent nitrite oxidation pathway (Eiserich et al. 1998).

The production of RNS was reported to be upregulated in the airways of asthmatic patients (Saleh et al. 1998; Hamid et al. 1993). Recently, we reported that the production of RNS was also upregulated in the COPD airways based on immunostaining for 3-nitrotyrosine (3-NT), which is a footprint of nitrosative stress (Ichinose et al. 2000). Since this method was semi-quantitative, we developed a new technique for the quantitative measurement of 3-NT which utilizes high-performance liquid chromatography with an electrochemical detection (HPLC-ECD) system. Using this system, we reported that the protein-bound 3-NT levels were increased in the induced sputum of both COPD and asthmatic patients, and there was a significant correlation between 3-NT/tyrosine value and % predicted forced expiratory volume in one second (FEV_1_) in COPD patients but not in asthmatic patients (Sugiura et al. 2004). However, there are some problems to be resolved to improve the accuracy of the measurement system.

We have focused on the measurement of protein-bound 3-NT (Sugiura et al. 2004; Hirano et al. 2006) because the nitration of tyrosine residues in various proteins was reported to alter the function of the proteins (Beckman et al. 1990; Radi et al. 1991; Lipton et al. 1993; Beckman, 1996). However, The various biological samples contain both protein-bound 3-NT and the free amino acid form of 3-NT (Greenacre and Ischiropoulos, 2001), and the influence of the amount of the free amino acid form of 3-NT on the results of protein-bound 3-NT has not been assessed.

Furthermore, though the induced sputum technique by hypertonic saline inhalation that we used is an effective and relatively noninvasive method for obtaining airway secretions (Pin et al. 1992; Fahy et al. 1993), this method may have the disadvantage of a considerable and unpredictable level of salivary contamination which could influence the values of 3-NT in the induced sputum, and this issue has not been fully addressed.

Therefore, the aims of this study were to investigate the influence of the free amino acid form of 3-NT on the measurement of protein-bound 3-NT, and to evaluate the influence of saliva on the measurement of protein-bound 3-NT in induced sputum by means of HPLC-ECD in COPD patients.

## Methods

### Study subjects

Five male patients with COPD visiting our hospital were recruited in the current study after providing written informed consent. All patients were diagnosed as COPD and satisfied the definition of the Global Initiative for Chronic Obstructive Lung Disease (National Institutes of Health, Updated 2005). The study was approved by the local ethics committee. The clinical characteristics of these subjects are shown in Table 1.

### Sputum induction and saliva sampling

Sputum was induced according to the method described in previous studies (Pin et al. 1992; Hirano et al. 2006). Briefly, to prevent the bronchoconstriction that would be induced by hypertonic saline inhalation, all subjects inhaled salbutamol (400 μg) before sampling. Fifteen minutes after salbutamol inhalation, the subjects inhaled 4% hypertonic saline using an ultrasonic nebulizer (UN-701; AICA Co Ltd, Tokyo, Japan). Sputum sampling was performed every five minutes until the sputum volume was more than 1.0 ml. In order to prevent saliva contamination in the sputum, the oral cavity was rinsed out with water, and retained saliva was removed by rolling the sputum on dry gauze. Saliva was also obtained after sputum induction. Over 1.0 ml of saliva was sampled.

### Sample processing

Sputum and saliva were immediately and gently treated with dithiothreitol (Oxoid Ltd, Basingstoke, Hampshire, UK) at 4 times the volume to dissociate the disulphide bonds in mucin molecules. The mixtures were centrifuged at 790 g for 5 min at 4°C and the supernatants were obtained. All supernatants were stored at −80°C until the measurement of 3-NT and tyrosine.

### Preparation of protein-bound 3-NT, tyrosine, and free 3-NT

The protein-bound 3-NT and tyrosine in sputum and saliva were measured by the protein hydrolysate according to the method reported in the previous study (Sugiura et al. 2004). Briefly, a supernatant sample was centrifuged at 9000 g for 5 min to remove impurities. The supernatant sample was centrifuged again at 9000 g for 30 min with an Ultrafree-MC centrifugal filter (Millipore Corp, Bedford, MA, USA) which can filtrate proteins of less than 10 kDa. The filtrate was prepared for the measurement of the free amino acid form of 3-NT, because the presence of protein of more than 10 kDa causes an obstruction in the HPLC-ECD column. The condensed supernatant was enzymatically hydrolyzed to liberate the 3-NT and tyrosine residues for the measurement of protein-bound 3-NT and tyrosine. Samples were mixed with a freshly prepared solution of *Streptomyces griseus* Pronase (Calbiochem, Darmstadt, Germany), which was dialyzed against phosphate buffered saline (pH 7.2) before use. The sample and pronase solution were mixed in a proportion of five to one in total protein. The pronase-treated samples were incubated at 50°C for 18 hours to hydrolyze the proteins. The hydrolysate was centrifuged at 9000 g with filtration for 30 min using an Ultrafree-MC centrifugal filter (10 kDa cut off), and then the filtrates were analyzed for 3-NT and tyrosine by HPLC-ECD. The protein concentration was determined by the Bradford method (Bradford, 1976).

### Quantification of 3-NT by HPLC-ECD

The sample was subjected to a reverse phase column (C18:3 × 150 mm; Eicom, Kyoto, Japan) and eluted under isocratic conditions with 100 mM sodium phosphate buffer (pH 5.0) containing 5% methanol at a flow rate of 0.5 ml/min. The eluate was continuously applied to the analytical electrochemical system that consisted of two electrochemical cells. The upstream electrochemical cell was coulometric and made of porous carbon. 3-NT was reduced into 3-aminotyrosine on this cell at a reduction potential of −900 mV. The downstream cell was amperometric glassy carbon to oxidize the 3-aminotyrosine at an oxidation potential of +300 mV. The 3-NT was quantified by the response at the oxidation cell on the basis of a standard curve of electrochemical responses as a function of the authentic 3-NT (Sigma Chemical Co, St Louis, MO) concentration. The specificity of the detection of the peak for 3-NT by this system was confirmed according to the previous study and the following criteria: [1]Comparison of the retention time of the peak with that of authentic 3-NT, which is 12.9 min under these HPLC-ECD conditions, [2]Disappearance of the peak after treatment of the sample with 100 mM sodium hydrosulfate (Na_2_S_2_O_4_) in PBS (pH 7.4) for 30 min at 37°C, [3] Nullification of the peak using a reduction potential of −600 mV (Sugiura et al. 2004). The values of concentration were expressed as those in original sputum or saliva.

### Quantification of tyrosine by HPLC-ECD

The amount of 3-NT in each sample was standardized by the amount of tyrosine in the same sample, which was determined in another HPLC-ECD system for tyrosine. The hydrolysate was injected into a reverse phase column (4.6 × 150 mm, TSK gel ODS-80TS; Tosoh, Tokyo, Japan) and eluted to separate tyrosine under isocratic conditions with 50 mM sodium acetate buffer (pH 4.7) plus 5% methanol at a flow rate of 0.8 ml/min. The tyrosine peak was detected using an HPLC-ECD system (D-7000, Hitachi, Tokyo, Japan) and electrochemical detector (NANOSPACE, Shiseido, Tokyo, Japan). The retention time of tyrosine under these conditions is 4.1 min and showed a typical profile of electrochemical responses at +600 mV (Sugiura et al. 2004). The values of the concentration were expressed as those in original sputum or saliva.

### Statistical analysis

The data are presented as mean ± SD. Mann-Whitney’s U test was used to compare the concentration of 3-NT or tyrosine in sputum and saliva. A *p*-value of less than 0.05 was considered significant.

## Results

### Standard curve and detection limit

**Figure 1.**
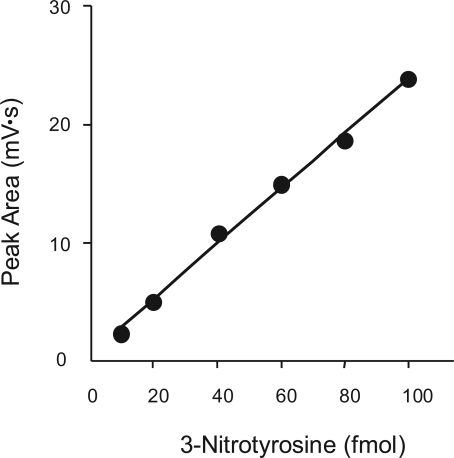
**Standard curve for 3-nitrotyrosine by high-performance liquid chromatography with electrochemical detection (HPLC-ECD).** A linear electrochemical response was observed in a wide range of authentic 3-nitrotyrosine concentrations. The detection limit of this HPLC-ECD system was 10 fmol.

We examined the dose dependent profile of the electrochemical response for authentic 3-NT. A linear electrochemical response was observed in a wide range of authentic 3-NT concentrations (10–1000 fmol in absolute amounts). Actually, the range of 3-NT in sputum and saliva in this system was up to 100 fmol from our preliminary data. The detection limit was 10 fmol (Fig. 1).

### Protein-bound and free 3-NT

**Figure 2.**
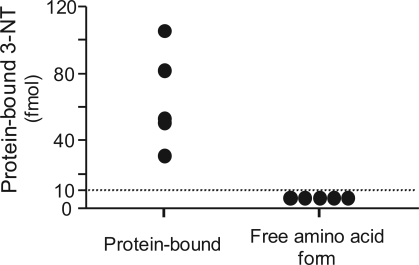
**The levels of protein-bound and the free amino acid form of 3-nitrotyrosine in the hydrolysate from COPD patients.** The amount of the free amino acid form of 3-nitrotyrosine was under the detection limit (<10 fmol) and much smaller than that of protein-bound 3-nitrotyrosine. 3-NT: 3-nitrotyrosine.

We analyzed the formation of the free amino acid form of 3-NT in supernatants of sputum by using filtrates obtained with an ultrafiltration tube (10 kDa cut off). The amounts of protein-bound 3-NT and the free amino acid form of 3-NT when 50 μl hydrolysate were injected are shown in Fig. 2. The mean value of the amount of protein-bound 3-NT was 65.0 fmol (31.2 to 106.4 fmol), whereas the amount of the free amino acid form of 3-NT was under the detection limit (<10 fmol) with this system (Fig. 2).

### Protein-bound 3-NT and tyrosine in sputum and saliva

**Figure 3.**
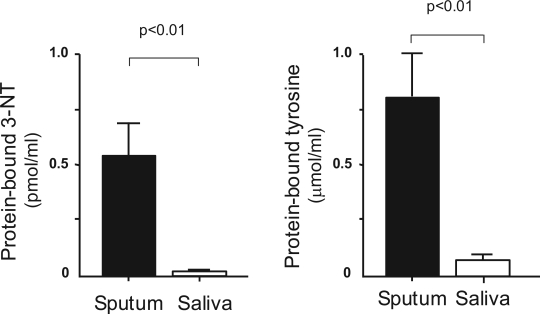
**Concentration of protein-bound 3-nitrotyrosine and protein-bound tyrosine in sputum and saliva.** Both the concentrations of protein-bound 3-nitrotyrosine and protein-bound tyrosine were significantly lower in saliva than in sputum. 3-NT: 3-nitrotyrosine. Data are presented as mean ± SD.

In COPD patients, the levels of 3-NT in saliva were significantly lower than those in sputum (sputum: 0.55 ± 0.15 pmol/ml, saliva: 0.02 ± 0.01 pmol/ml; *p* < 0.01). The levels of tyrosine in saliva were also significantly lower than in sputum (sputum: 0.81 ± 0.43 micro mol/ml, saliva: 0.07 ± 0.04 micro mol/ml; *p* < 0.01) (Fig. 3). The values of 3-NT and tyrosine in saliva were about 3.1% and 9.0% of those in sputum, respectively.

## Discussion

In the current study, we found that the amount of free amino acid form of 3-NT was very small and was under the detection limit in this HPLC-ECD system, though it could be present in the sputum. This suggests that the influence of the free amino acid form of 3-NT on the values of protein-bound 3-NT would be very small and could be negligible. Furthermore, we found that the values of 3-NT and tyrosine in saliva were 3.1%, 9.0% of those in sputum, respectively.

Tyrosine is one of the amino acids that compose proteins. Protein-bound 3-NT and protein-bound tyrosine are composed of various proteins, and free amino acid forms of 3-NT and of tyrosine are present in induced sputum. The free amino acid form of 3-NT might affect the measured values of protein-bound 3-NT by HPLC-ECD. Compared with the amount of protein-bound 3-NT, which was 65.0 fmol, that of the free amino acid form of 3-NT was very small and under the detection limit (<10 fmol). This suggests that the influence of the contamination of free 3-NT would be very small.

Salivary contamination in induced sputum cannot be avoided completely, but most can be removed by gargling and then by rolling the sputum samples on dry gauze as much as possible after the induction. To reduce the contribution of saliva to the induced sputum samples, sputum and saliva have been collected separately during sputum induction for measuring cytokines (Keatings et al. 1996) or eosinophil cationic protein (ECP) (Gershman et al. 1996). Pin et al. restricted their analyses of induced sputum to plugs of mucus extracted from the sample, and the volume of the contaminating saliva was assumed to be very small in comparison with the volume of sputum (Pin et al. 1992). These reports suggest that the contribution of saliva to the measurement values can be minimized by various methods.

Examinations of the influence of salivary contamination in induced sputum have been reported. Fahy et al. (Fahy et al. 1993) reported that the amount of ECP in saliva from asthmatic subjects was much lower than in paired induced sputum samples. They concluded that the principal effect of saliva in induced sputum was only that of diluting the sample. Dauletbaev et al. (Dauletbaev et al. 2001) reported that the glutathione content of the saliva was significantly lower than that in the sputum. Sagel et al. (Sagel et al. 2001) reported that the salivary IL-8 levels were one-fifteenth the levels in the induced sputum samples and were, therefore, unlikely to significantly affect the levels measured in the sputum. These reports were compatible with our data, in which the concentrations of 3-NT and tyrosine in saliva were 3.1% and 9.0% of those in induced sputum, respectively.

For the measurement of 3-NT from human samples, a large number of studies using antibody-based methods have been reported. For example, immunohistochemical staining of 3-NT has been reported in the tissue or fluid from inflamed colonic epithelium (Singer et al. 1996), chronic hepatic diseases (Cuzzocrea et al. 1998; Garcia-Monzon et al. 2000), atherosclerotic plaques (Cromheeke et al. 1999), Alzheimer’s disease (Smith et al. 1997) and cystic fibrosis (Morrissey et al. 2002). We also measured the 3-NT in induced sputum from COPD patients by immunohistochemical staining (Ichinose et al. 2000; Hirano et al. 2006), but this method is semi-quantitative. Other measurements made by employing ELISA assay have been reported (Ceriello et al. 2001; Oldreive et al. 2001; ter Steege et al. 1998; Banks et al. 1998), but there was no rigorous assay validation and the reliability was poor because the peroxidase employed in the process of ELISA might have stimulated the production of 3-NT (Duncan, 2003).

HPLC combined with an electrochemical detection method is a quantitative method for measuring 3-NT. Both UV (Crow and Ischiropoulos, 1996) and fluorescence detection (Kamisaki et al. 1996) have been employed, but they were too insensitive. Consequently, HPLC-EC systems have been commonly employed to measure 3-NT in human samples (Crow, 1999; Hensley et al. 1998; Moore and Mani, 2002; Shigenaga et al. 1997; Ohshima et al. 1999). In these studies, the detection limit of 3-NT was 0.1 pmol/20 μl injection, but with our current HPLC-ECD system the detection limit was 10 fmol/10 μl injection which was 20 times more sensitive than the other reported methods. The current HPLC-ECD is thought to be the most accurate system for quantifying 3-NT in sputum.

We previously reported that the levels of 3-NT were increased in induced sputum from asthma and COPD patients by HPLC-ECD (Sugiura et al. 2004), and that theophylline reduced the levels of 3-NT in COPD patients (Hirano et al. 2006). Our present report supports the precision of the data obtained with this HPLC-ECD system.

The presence of co-morbid diseases associated with higher protein-bound 3-NT production in saliva such as periodontal disease, might influence the values of 3-NT in sputum. Further study should be necessary to clarify the influence of co-morbid diseases in oral cavity.

In conclusion, the free amino acid form of 3-NT does not affect the measurement of protein-bound 3-NT. Furthermore, the influence of salivary contamination on the measurement of protein-bound 3-NT in induced sputum by means of HPLC-ECD is very small and could be negligible.

## Figures and Tables

**Table 1.** Characteristics of Study Subjects.

Sex (M/F)	5/0
Age (years)	71.6 ± 2.4
Smoking	
current/ex	5/0
pack-year	69.2 ± 20.4
FEV_1_ (L)	1.72 ± 0.80
FEV_1_/FVC (%)	45.0 ± 14.5
FEV_1_%predicted (%)	64.4 ± 29.5
IC (L)	2.83 ± 0.59

Definition of abbreviations: FEV1 = forced expiratory volume in one second, FVC = forced vital capacity, IC = inspiratory capacity, Data are expressed as mean ± SD
